# New York State Veterinary Medical Society

**Published:** 1893-01

**Authors:** R. S. Huidekoper, N. P. Hinkley

**Affiliations:** President; Secretary


### NEW YORK STATE VETERINARY MEDICAL SOCIETY.

The Secretary calls your attention to the next and "Third Annual Meeting of the New York State Veterinary Medical Society," which will meet at the assembly room of the Vanderbilt

House, Syracuse, N. Y., on Thursday, January 12th, 1893, at 10 o'clock A.M. *sharp.*

At this meeting we want to excel all others by having the largest attendance, and most interesting debates on the subjects to be brought before the members. We will also have the annual election of officers and the report of the Committee on the Revision of the Constitution and By-laws. The work of this Committee alone should call out every member of the profession to debate and express his thoughts, and vote on the proposed changes in so important a matter as revision of the Constitution and By-laws. The report of the Committee on Legislation will be submitted, also that of the Secretary and Treasurer for the past year. As Veterinarians we must make arrangements at this meeting to appoint delegates to attend the First International Congress of Veterinarians, to be held in Chicago in September, 1893. to properly represent the New York State Veterinary Medical Society at this congress.

By consulting the programme you will find that we have some very important subjects for discussion, including the question of changing the place of meeting, and to decide which will be preferable, a *one day's session semi-annually* or a *two days' session annually.*

*Order of Business:*—Roll Call; Reading of Minutes of Last Meeting; Address by President R. S. Huidekoper; Receiving Applications for Membership; Examination of Applications by the Censors; Report of Censors on Applications; Introduction of New Members; Report of the Secretary; Unfinished Business; New Business; Communications and Correspondence; Essays and Papers; Discussion of Essays and Papers; Report of the Treasurer; Election of Officers; Adjournment.

*Reports of Committees:*—Report of Committee on Constitution and By-laws, Dr. R. A. McLean, Chairman. Report of Committee on Legislation, Dr. C. D. Morris, Chairman. Report of Board of Censors, Dr. John A. Bell, Chairman. Report of County Secretaries. Report of Committee on Publications, Dr N. P. Hinkley, Chairman. Report of Committee on Arrangements, Dr. N. P. Hinkley, Chairman. Subjects for Discussion: "Influenza, and its Complications;" "Antiseptics; their Uses and Modes of Application;" "Tuberculosis, especially its Generation."

You are cordially invited to be present at this meeting. By order of DR. R. S. HUIDEKOPER, President.

DR. N. P. HINKLEY, Secretary.

